# Age-Dependent Efficacy of Incidental Coronary Artery Calcium on Pre-CAG Chest CT: Potentially Missed Prevention Opportunities in Chinese Population

**DOI:** 10.3390/jcdd13090471

**Published:** 2026-09-20

**Authors:** Sang Zhou, Yufei Guo, Lili Wang, Chengzhu Wang, Zhitao Jin

**Affiliations:** PLA Rocket Force Characteristic Medical Center, Beijing 100088, China; sang_zhou_cardiomed@outlook.com (S.Z.); yufeiguo_cardio@outlook.com (Y.G.); lili_wang_cardiomed@outlook.com (L.W.)

**Keywords:** coronary artery calcium, non-ECG-gated chest CT, coronary angiography, obstructive coronary artery disease, artificial intelligence, cardiovascular primary prevention

## Abstract

The 2026 ACC/AHA Multisociety Dyslipidemia Management Guideline emphasizes the clinical utility of coronary artery calcium (CAC) and recommends integrating incidental CAC from non-ECG-gated chest CT into cardiovascular risk-stratification workflows; nevertheless, such incidental findings remain substantially underutilized in routine clinical practice. Many young-to-middle-aged patients present with acute myocardial infarction as their index cardiovascular event, even though prior chest CT could identify CAC that would trigger statin-based primary prevention. This single-center retrospective observational study enrolled 976 patients undergoing invasive coronary angiography (CAG) who had non-ECG-gated chest CT within the preceding 1 year (2020–2025) to assess cross-sectional associations between incidental CAC burden and angiographic coronary stenosis. CAC burden was categorized as Grade 0–3 using the validated Shemesh ordinal scoring system, with 79.2% of participants being CAC-positive. CAC positivity and obstructive CAD prevalence increased progressively with age. CAC positivity was associated with higher conditional odds of obstructive CAD among younger individuals, while this conditional risk signal was attenuated in older adults. CAC grade showed a step-wise positive correlation with stenosis severity. Following adjustment for age, sex, hypertension, diabetes mellitus, dyslipidemia, and smoking status, CAC was identified by multivariate regression as the strongest independent predictor of obstructive CAD (OR = 11.732, 95% CI: 7.700–17.875, *p* < 0.001). Among CAD patients, CAC-negative subjects had higher diabetes prevalence, in keeping with the propensity for non-calcified vulnerable plaques in diabetic individuals. Incidental CAC on routine chest CT represents a robust age-modulated biomarker for obstructive CAD, and considerable primary-prevention opportunities are missed in Chinese patients referred for CAG. Opportunistic age-stratified CAC screening assisted by artificial intelligence may improve real-world cardiovascular primary prevention.

## 1. Introduction

Atherosclerotic cardiovascular disease (ASCVD) remains the leading global cause of mortality, with obstructive coronary artery disease (CAD) acting as the primary pathological driver of acute myocardial infarction (AMI) and sudden cardiac death [1]. The 2026 ACC/AHA Multisociety Guideline on the Management of Dyslipidemia substantially elevates the clinical value of coronary artery calcium (CAC), designating CAC assessment a Class I recommendation for re-stratifying ASCVD risk. The guideline explicitly recommends statin-initiated lipid-lowering therapy regardless of baseline lipid profiles for individuals with mild CAC (Agatston score 1–99) or CAC burden exceeding the age-specific 75th percentile [2]. Despite these guideline recommendations, electrocardiogram (ECG)-gated coronary computed tomographic angiography (CCTA)—the gold-standard modality for quantitative CAC measurement—has limited routine clinical application, leaving most asymptomatic individuals without standardized CAC risk evaluation. In contrast, non-ECG-gated chest CT is widely used across China for non-cardiac clinical indications, including follow-up of pulmonary infection, lung nodule surveillance, thoracic trauma evaluation, and routine health screening. The 2026 ACC/AHA dyslipidemia guideline specifies that incidental CAC detected on non-ECG-gated chest CT constitutes objective evidence of subclinical atherosclerosis and must be incorporated into all cardiovascular diagnostic and therapeutic decision-making workflows [2]. Regrettably, incidental CAC identified on routine non-ECG-gated chest CT is vastly underrecognized and underutilized in routine clinical practice in China.

As illustrative real-world clinical examples, we observed several men aged around 40 years at our Beijing-based tertiary hospital who suffered de-novo AMI in the absence of preceding prodromal ischaemic symptoms such as chest tightness or angina pectoris. Retrospective review of their prior screening imaging revealed definite CAC lesions on earlier noncontrast non-ECG-gated chest CT scans, with a 1–3 year interval between initial CAC detection and AMI onset. This time window represented an ample opportunity to implement statin-mediated primary cardiovascular prevention in accordance with the updated guideline recommendations. To date, no retrospective observational studies targeting Asian populations have explored incidental CAC detected on pre-procedural noncontrast non-ECG-gated chest CT among patients undergoing coronary angiography (CAG). To address this research gap, we performed a single-center retrospective observational analysis grounded in the framework of the 2026 ACC/AHA Multisociety Guideline on the Management of Dyslipidemia. Using real-world clinical data from China, we assessed the clinical utility of opportunistic CAC screening via non-ECG-gated chest CT. We particularly focused on the age-stratified diagnostic performance of visual Shemesh CAC grading within this Chinese CAG-referred population and quantified the extent of missed statin-mediated primary-prevention opportunities. This study further addresses three core clinical questions: the proportion of patients with CAD who exhibited CAC warning signs on prior chest CT but received no preventive interventions, residual CAD risk among individuals with CAC-negative scans, and the clinical characteristics of patients with obstructive CAD despite undetectable CAC.

## 2. Materials and Methods

### 2.1. Study Design and Patients

This retrospective single-center observational study consecutively enrolled patients who received invasive coronary angiography (CAG) for suspected new-onset coronary artery disease (CAD) between 1 January 2020 and 31 December 2025. Patients meeting any of the following criteria were excluded: (1) those with a prior history of percutaneous coronary intervention (PCI) or coronary artery bypass grafting (CABG); (2) patients who had no preoperative non-ECG-gated chest CT examination; (3) individuals with an interval longer than 1 year between non-ECG-gated chest CT and CAG. The examination interval was limited to ≤1 year to minimize confounding bias caused by interval progression of coronary atherosclerotic plaques. A longer time gap would raise the possibility of new calcification or plaque progression occurring after chest-CT acquisition, decoupling CAC findings from CAD severity identified at subsequent CAG. Most non-ECG-gated chest CT scans were acquired within one month prior to invasive angiography. While this narrow time window strengthens the alignment between CAC findings and angiographic coronary lesions and reduces confounding from interval plaque evolution, it limits our ability to draw causal temporal inference on whether CAC precedes the development of obstructive stenosis. Accordingly, our analysis reflects cross-sectional associations rather than prospective prognostic cohort evidence. The study protocol was approved by the Institutional Review Board of our hospital. Written informed consent was waived due to the retrospective study design.

### 2.2. Data Collection

Baseline demographic and laboratory indicators including age, sex, and body mass index (BMI) were collected from routine clinical examinations performed before coronary angiography (CAG). Current smoking was defined as cigarette consumption within the preceding 30 days. Hypertension was diagnosed based on systolic blood pressure ≥ 140 mmHg, diastolic blood pressure ≥ 90 mmHg, or regular use of antihypertensive agents. Diabetes mellitus was confirmed by a self-reported medical history of diabetes, ongoing anti-diabetic medication use, or fasting blood glucose ≥ 126 mg/dL. Dyslipidaemia was identified according to one of the following two criteria: (1) clearly documented long-term administration of lipid-lowering agents, irrespective of inpatient lipid laboratory results, or (2) laboratory-verified elevated lipid levels on inpatient testing, defined as low-density lipoprotein cholesterol > 155 mg/dL or reduced high-density lipoprotein cholesterol (<40 mg/dL for males and <50 mg/dL for females). For participants without explicit documentation of prior lipid-lowering medication use, dyslipidaemia status was determined solely based on inpatient laboratory lipid values, and no imputation of unrecorded prior lipid-lowering-drug exposure was performed. All clinical data involved in this study were extracted from the hospital electronic medical record system starting on 10 February 2026 (data collection period: 10 February 2026–25 February 2026).

### 2.3. CAC Grading and Image Interpretation

Thin-slice non-ECG-gated chest scans were performed using 256-slice or higher multi-detector computed tomography (MDCT). Images were reconstructed with a standard soft-tissue kernel, with reconstructed slice thickness and interval set at 2.5 mm. Minor adjustments to scanning parameters were permitted according to patient-specific factors such as body habitus. No cardiac-specific scanning protocol was implemented. Quantitative visual scoring was conducted according to the grading system established by Shemesh et al. [3]. The four coronary segments evaluated included the right coronary artery, left main coronary artery, left anterior descending artery, and left circumflex artery. Each coronary vascular segment was assigned a score of 0 to 3 according to the longitudinal range of calcified lesions: a score of 0 indicated the absence of coronary calcification; a score of 1 represented calcification occupying less than one-third of the total vessel length; a score of 2 referred to calcification involving one-third to two-thirds of the vessel length; and a score of 3 denoted calcification extending over two-thirds or more of the vessel length. The total CAC score was calculated as the sum of individual scores from all four vascular segments, with a total score ranging from 0 to 12 points. Based on the summed score, overall coronary atherosclerotic burden was stratified into four grades: Grade 0 for a total score of 0 corresponding to no calcification, Grade 1 for scores of 1 to 3 indicating mild calcification, Grade 2 for scores of 4 to 6 defined as moderate calcification, and Grade 3 for scores of 7 to 12 representing severe calcification. All CT images were independently and blindly reviewed by two senior radiologists. Inter-observer agreement for the Shemesh CAC scoring was assessed using Cohen’s Kappa coefficient, with a κ value of 0.81 (95% CI: 0.76–0.87), indicating excellent inter-reader consistency. Discrepancies between the two readers were resolved by final adjudication from a senior radiologist.

### 2.4. Coronary Angiography

Coronary angiography (CAG) was performed via the standard Judkins technique. At least two orthogonal projections were obtained for the right coronary artery (RCA), and a minimum of five projections were acquired for the left coronary arterial system. All angiographic images were interpreted independently by two experienced interventional cardiologists. Stenosis severity was categorized as normal (0% stenosis), luminal narrowing <50%, 50–69%, and ≥70% up to total occlusion. Coronary artery disease (CAD) was defined as stenosis ≥ 50% in at least one major epicardial coronary vessel. Coronary stenosis was assessed by visual-only anatomic evaluation, without adjunctive functional testing such as fractional flow reserve.

### 2.5. Statistical Analysis

Statistical analyses were conducted using SPSS version 22.0 software (IBM Corporation, Armonk, NY, USA). A two-tailed *p* value less than 0.05 was set as the threshold for statistical significance. Continuous variables were expressed as mean ± standard deviation, while categorical variables were presented as case number and corresponding percentage. Between-group comparisons of continuous variables were carried out using the independent Student’s *t*-test. The Chi-square (χ^2^) test or Fisher’s exact test was adopted for categorical variables when the theoretical frequency was less than 5. The linear-by-linear association test was applied to detect the changing trend across age subgroups. Stratified analyses were performed to compare the prevalence of CAD and different grades of coronary stenosis among subgroups stratified by CAC grades, so as to clarify the association between CAC burden and CAD. Multivariate binary logistic regression was constructed to screen independent predictive factors for CAD, with the Hosmer–Lemeshow goodness-of-fit test used to evaluate model calibration. The Wald χ^2^ test was utilized to assess the predictive performance of the regression model for coronary stenosis ≥ 50% and ≥70%. Multinomial logistic regression was further applied to investigate the dose–response relationship between CAC grading and the severity of coronary stenosis. Age-stratified trend analyses were pre-specified exploratory secondary analyses using linear-by-linear association tests; exhaustive pairwise comparisons across all age subgroups were not performed, and formal multiple-testing correction was therefore not applied.

## 3. Results

### 3.1. Patient Characteristics

**Figure 1:**
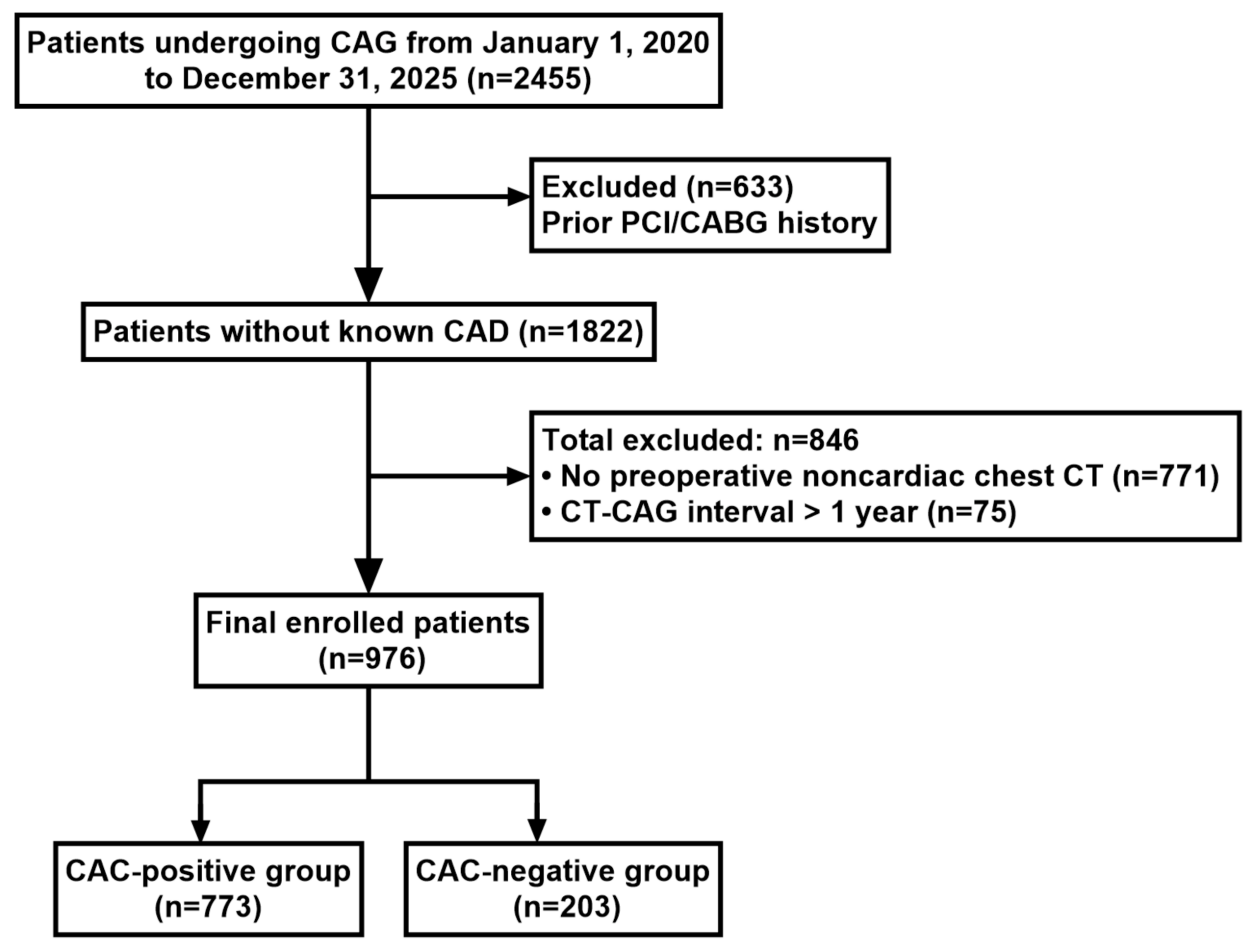
Study flow diagram.

From January 2020 to December 2025, a total of 2455 patients underwent invasive coronary angiography (CAG) at the Department of Cardiology of our hospital. Of these individuals, 846 patients were excluded from the analysis: 633 had a prior history of percutaneous coronary intervention (PCI) or coronary artery bypass grafting (CABG), 771 failed to complete preoperative non-ECG-gated chest computed tomography (CT), and 75 exhibited an interval exceeding 1 year between CT acquisition and CAG (Figure 1). The remaining 976 eligible participants were enrolled for final statistical analysis and stratified into the CAC-positive group (*n* = 773) and CAC-negative group (*n* = 203) based on the presence of coronary artery calcification (CAC) (Figure 1).

Among the overall cohort, 78.1% (762/976) of patients had an interval of ≤3 months between non-ECG-gated chest CT and CAG, and 72.2% received CT scanning within 1 month prior to angiography. This narrow temporal window minimized confounding bias stemming from progressive coronary atherosclerotic plaque burden during the inter-examination period.

Baseline demographic, clinical and laboratory parameters of the two study groups are summarized in Table 1. Compared with patients in the CAC-negative group, those with detectable CAC were significantly older (65.63 ± 10.36 years vs. 60.23 ± 12.07 years, *p* < 0.001) and comprised a substantially higher proportion of male individuals (69.5% vs. 52.2%, *p* < 0.001). With regard to comorbidities and cardiovascular risk behaviors, the CAC-positive group demonstrated significantly higher prevalence of hypertension (67.7% vs. 58.1%, *p* = 0.011), diabetes mellitus (45.9% vs. 24.1%, *p* < 0.001), dyslipidemia (73.1% vs. 53.2%, *p* < 0.001), and active or historical smoking (46.4% vs. 28.1%, *p* < 0.001). In terms of laboratory biomarkers, fasting plasma glucose (6.30 ± 2.24 mmol/L vs. 5.80 ± 1.79 mmol/L, *p*= 0.001) and glycated hemoglobin (HbA1c; 6.52 ± 1.36% vs. 6.14 ± 1.45%, *p* = 0.001), were all markedly elevated in the CAC-positive cohort. No intergroup differences were observed in body mass index (BMI), LDL-C or triglyceride (TG) levels.

Notably, among the 1822 patients undergoing their first diagnostic coronary angiography with no prior history of coronary artery disease, 415 patients were hospitalized with acute myocardial infarction as the inaugural manifestation of CAD. Of these AMI patients, 55.7% (231 patients) completed preoperative non-ECG-gated chest CT within 1 year prior to angiography, including 106 cases of ST-segment elevation myocardial infarction (STEMI) and 125 cases of non-ST-segment elevation myocardial infarction (NSTEMI). Strikingly, CAC positivity was identified in 216 (93.5%) of these 231 patients who received CT scanning.

### 3.2. Trends in CAC-Positive Rate and CAD Prevalence by Age Group

**Figure 2:**
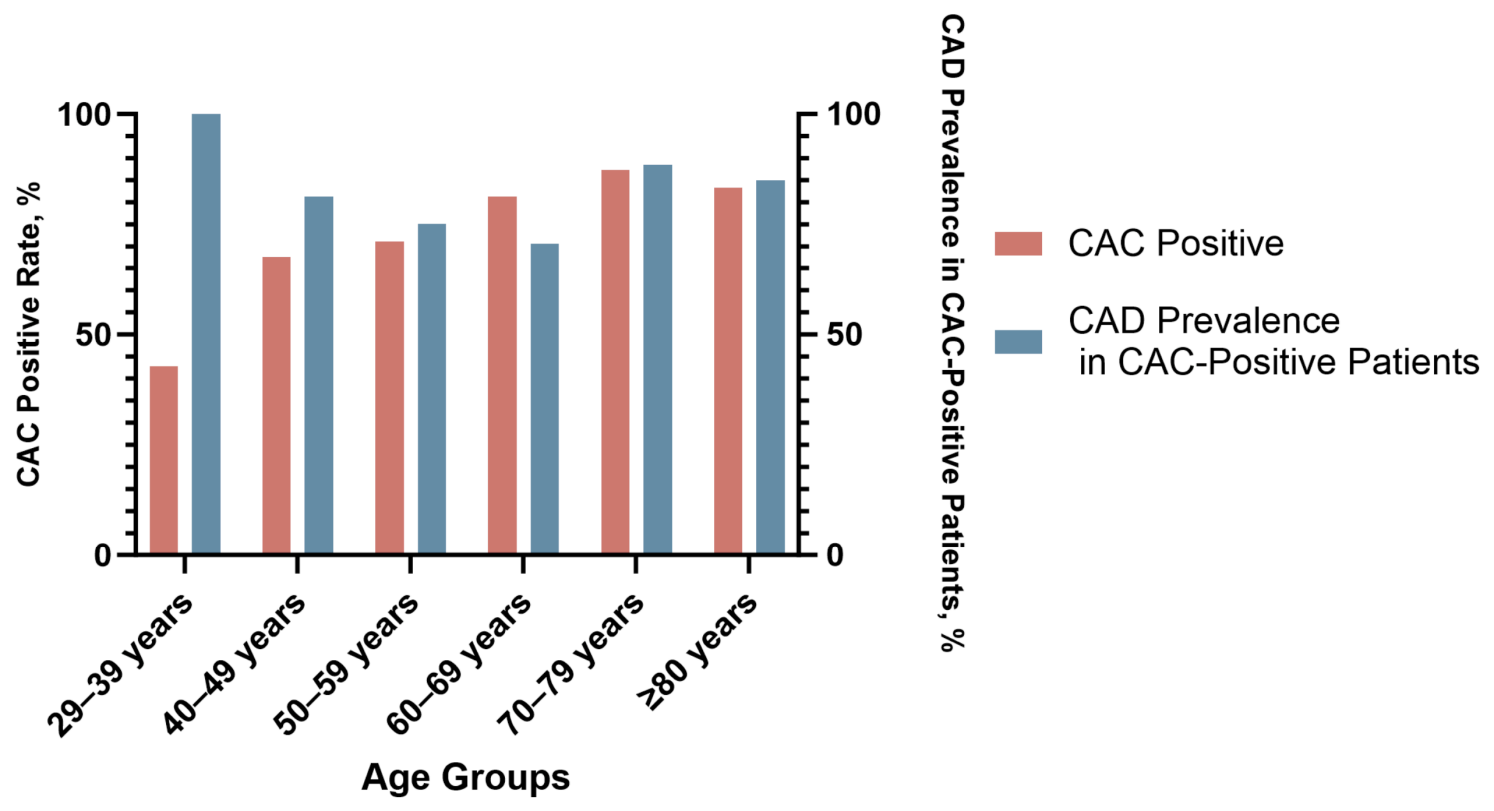
Age-stratified CAC-positive rate and CAD prevalence in CAC-positive patients.

Age-stratified trends for overall coronary artery calcification (CAC) positivity and coronary artery disease (CAD) prevalence within CAC-positive patients are displayed (Figure 2). CAC positivity increased linearly and significantly with advancing age, while CAD prevalence showed a marginally significant upward trend. CAC rates differed significantly across age groups (Pearson χ^2^ = 41.165, *p* < 0.001), with a positive linear association confirmed by linear-by-linear testing (χ^2^ = 37.435, *p* < 0.001). CAD prevalence among the 773 CAC-positive individuals also varied significantly by age (Pearson χ^2^ = 30.771, *p* < 0.001), with a marginally significant rising linear trend (χ^2^ = 3.754, *p* = 0.053). CAC was highly prevalent in patients aged ≥70 years with naturally high baseline CAD risk. In patients younger than 70, CAC positivity rose with age, yet younger subgroups carried higher CAD risk after CAC detection. The 29–39-year-old stratum demonstrated 100% CAD prevalence among CAC-positive patients; however, this finding is limited by the very small sample size of only nine subjects and potential selection bias inherent to our symptomatic CAG-referred cohort.

One cell (10.0%) had an expected frequency less than 5, and likelihood ratio tests produced consistent significant results (all *p* < 0.001), supporting robust trend findings. In short, aging is a key risk factor for CAC. Although CAC is widespread in elderly patients with high baseline CAD risk, CAC positivity bears greater clinical predictive value in younger populations, where it signals substantially higher odds of obstructive coronary disease. The distribution of CAC grades across age strata is provided in Supplementary Table S1.

### 3.3. Trends in Coronary Stenosis Risk Stratified by CAC Grades

**Figure 3:**
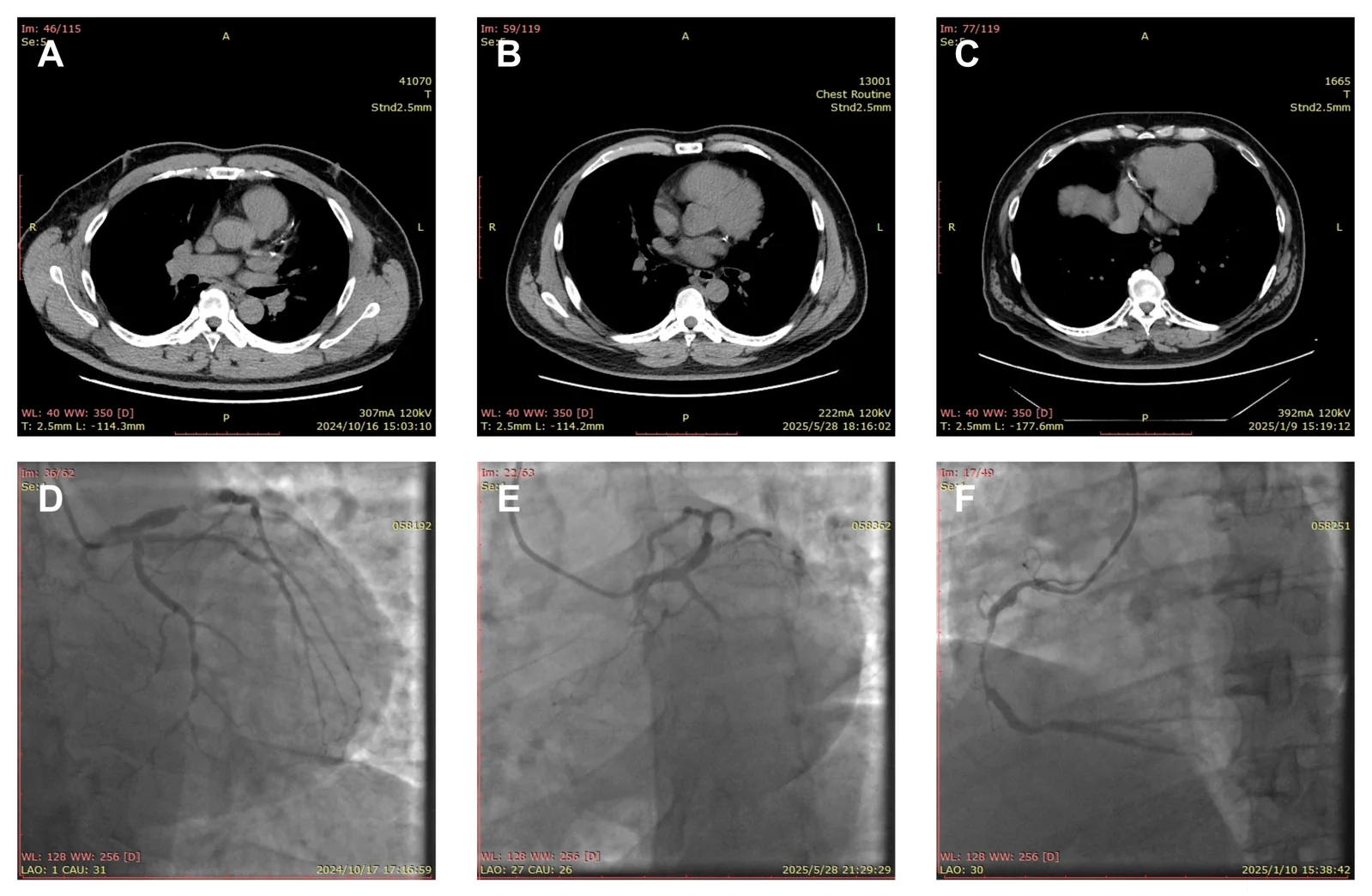
Representative paired imaging findings from three separate patients, showing incidental coronary artery calcification and matched obstructive coronary stenosis. Panels (**A**–**C**) display incidental calcification of LAD, LCX and RCA on non-ECG-gated routine chest CT, respectively. Panels (**D**–**F**) present the corresponding obstructive stenotic lesions of LAD, LCX, and RCA identified on invasive coronary angiography. Case matching: (**A**,**D**) originate from the same patient with LAD lesions; (**B**,**E**) belong to one patient with LAD lesions; (**C**,**F**) are acquired from a third patient with RCA lesions.

**Figure 4:**
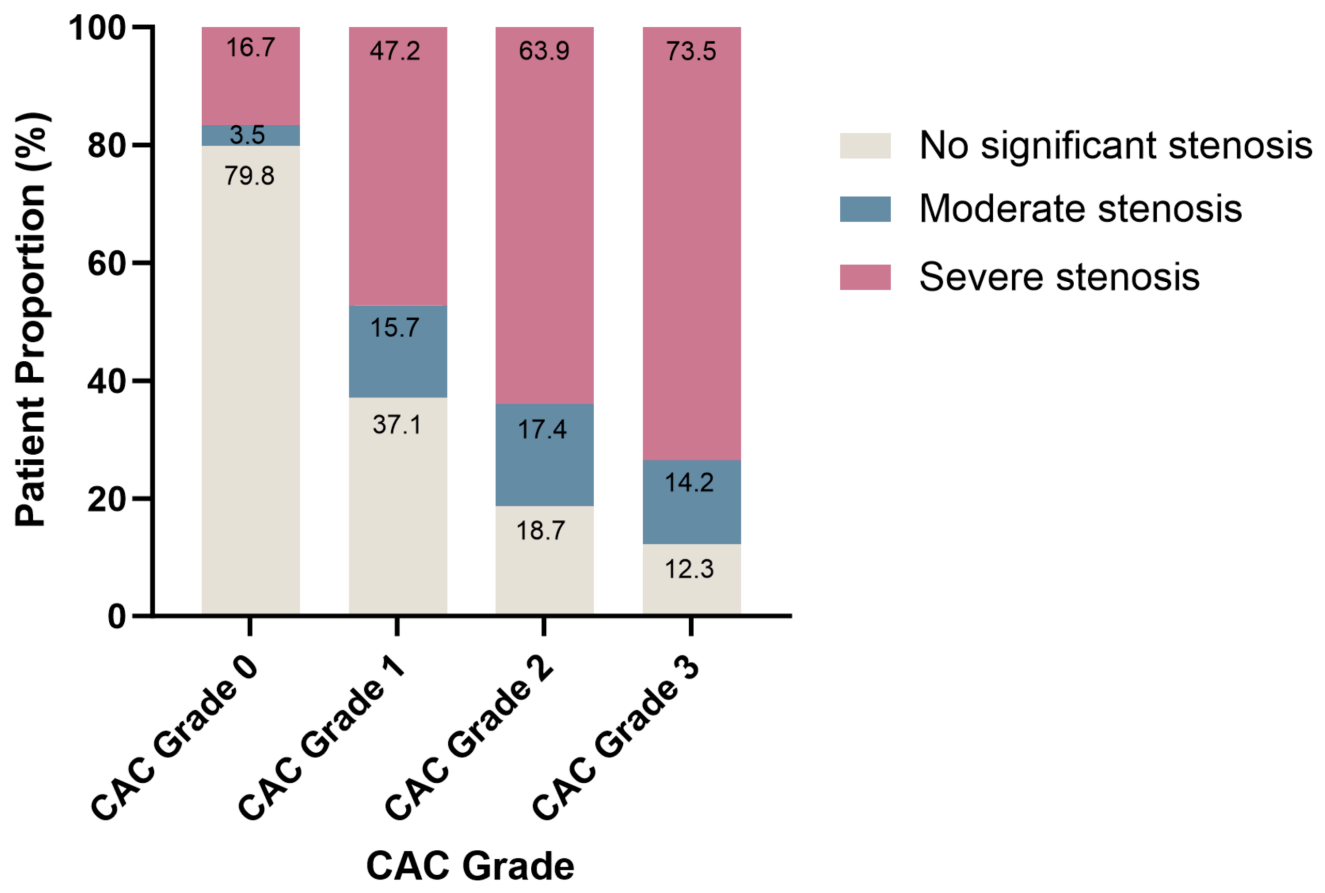
Distribution of coronary stenosis severity across four CAC grading strata. Stacked bar chart showing the proportional distribution of coronary stenosis severity stratified by CAC grades. With the elevation of CAC grade from 0 to 3, the proportion of patients with non-significant stenosis (<50% diameter stenosis) decreased progressively from 79.8% to 12.3%, while the proportion of severe stenosis (≥70% diameter stenosis) increased sharply from 16.7% to 73.5%. Moderate stenosis accounted for a relatively small fraction across all CAC subgroups.

Representative paired images showing incidental coronary artery calcification on routine non-ECG-gated chest CT and matched obstructive stenotic lesions confirmed by invasive coronary angiography are presented (Figure 3). The prevalence of obstructive coronary artery disease was markedly higher in CAC-positive patients than in CAC-negative individuals (*p* < 0.001). Stratified analysis demonstrated a graded upward trend in obstructive stenosis risk alongside elevated CAC grades (Table 2). The component ratio of different stenosis severities changed substantially across CAC strata, with a continuous decline in non-significant stenosis and a steep rise in severe stenosis as CAC grade increased (Figure 4).

Binary logistic regression analysis, with CAC Grade 0 set as the reference, confirmed that higher CAC grades were independently associated with significantly elevated odds of both ≥ 50% clinically significant stenosis and ≥70% severe stenosis (all *p* < 0.001). Multinomial logistic regression further verified the strong predictive performance of CAC grading for stenosis severity (Wald χ^2^ = 215.036 for ≥50% stenosis, Wald χ^2^ = 146.313 for ≥70% stenosis, all *p* < 0.001), and the Hosmer–Lemeshow test indicated satisfactory goodness of fit for the regression model (*p* > 0.05).

As illustrated in Figure 4, 79.8% of CAC-negative (Grade 0) participants exhibited non-significant coronary stenosis (<50% luminal narrowing) within this symptomatic CAG-referred cohort. This proportion represents a purely descriptive observation rather than a formal diagnostic-test negative predictive value applicable to general populations. It highlights that a meaningful subset of patients without detectable coronary calcification still harbour obstructive coronary lesions, consistent with the well-recognized tendency for non-calcified vulnerable plaques, particularly among patients with diabetes mellitus.

### 3.4. Independent Predictors of Coronary Artery Disease

**Figure 5:**
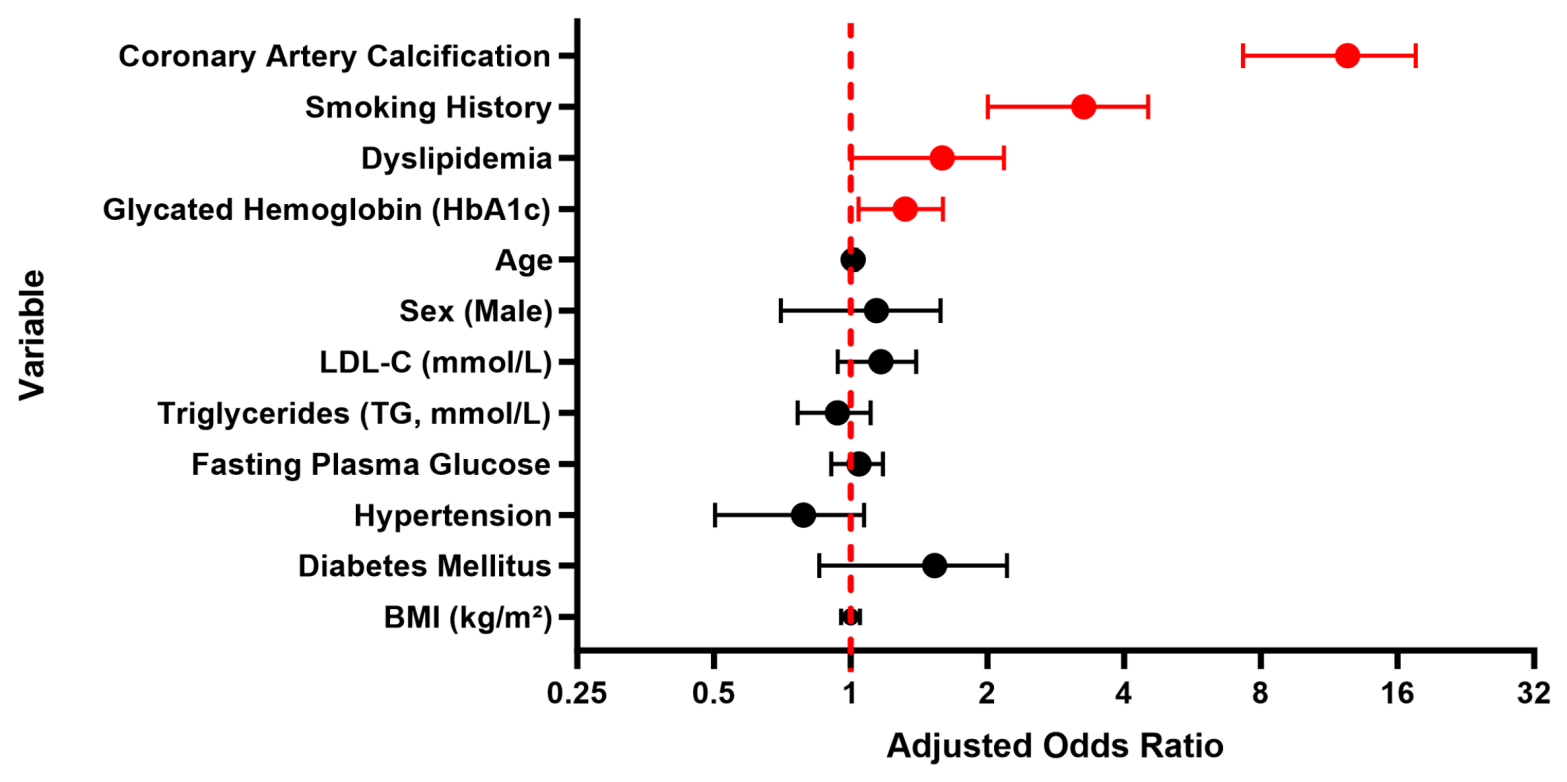
Forest plot of independent predictors for coronary artery disease from multivariate binary logistic regression. The vertical dashed line at OR = 1 represents the null effect. Factors whose 95% CIs do not cross the line were significant independent predictors (marked in red). Coronary artery calcification yielded the largest adjusted OR, indicating its powerful predictive value for CAD.

After comprehensive adjustment for traditional cardiovascular risk factors, the presence of coronary artery calcification (CAC) was independently associated with a markedly increased risk of coronary artery disease (CAD) (OR = 11.732, 95% CI: 7.700–17.875, *p* < 0.001). Of note, given the high prevalence of obstructive CAD (66.0%) within this symptomatic CAG-referred cohort, odds ratios from logistic regression will overestimate the magnitude of relative prevalence. Therefore, we report odds ratios solely for comparability with the prior cardiovascular literature and avoid interpreting these values as direct measures of relative disease risk. Other significant independent predictors of CAD included smoking history (OR = 3.102, 95% CI: 2.093–4.597, *p* < 0.001), dyslipidaemia (OR = 1.519, 95% CI: 1.045–2.209, *p* = 0.028), and elevated glycated haemoglobin (HbA1c) (OR = 1.300, 95% CI: 1.052–1.607, *p* = 0.015) (Figure 5, Table 3).

Notably, these cardiovascular risk factors demonstrated prominent synergistic effects on CAD risk. Patients with concomitant diabetes mellitus, dyslipidaemia, and hypertriglyceridaemia exhibited a 5.8-fold higher risk of CAD. In particular, smokers with CAC had a 52.2-fold increased CAD risk relative to individuals without a smoking history and CAC.

### 3.5. Comorbidity Profiles of CAD Patients Stratified by CAC Status

**Figure 6:**
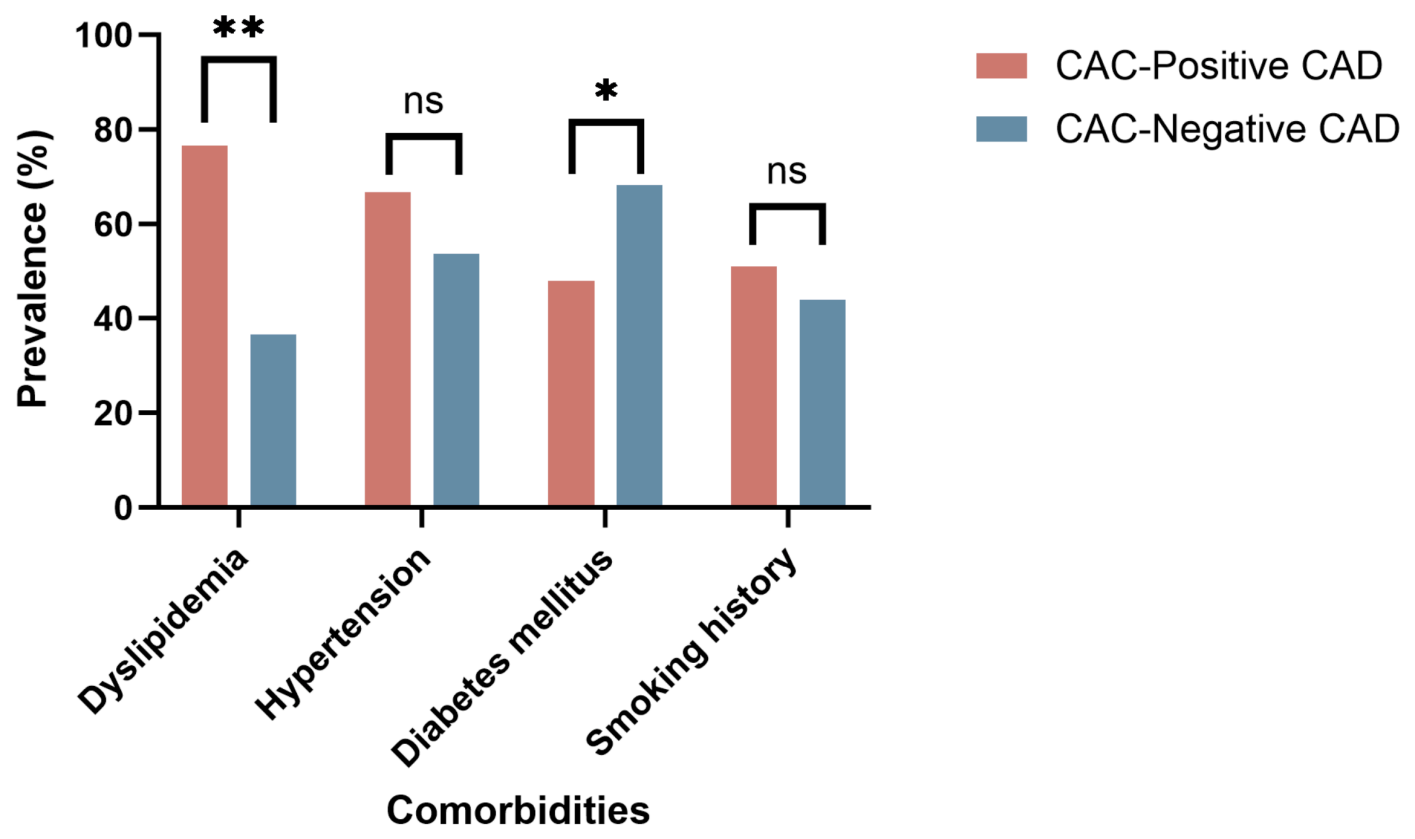
Comparison of comorbidity prevalence between CAC-positive and CAC-negative patients with coronary artery disease (CAD). Between-group differences were analysed using the Pearson’s chi-square test. ** *p* < 0.001, * *p* < 0.05, ns = non-significant.

Significant differences in the distribution of comorbidities were observed between CAD patients with positive CAC (*n* = 603) and those with negative CAC (*n* = 41) (Figure 6). The CAC-positive group presented higher prevalence rates of dyslipidaemia (76.5% vs. 36.6%, χ^2^ = 31.642, *p* < 0.001). No significant inter-group differences were found for hypertension (66.8% vs. 53.7%, χ^2^ = 2.969, *p* = 0.085) and smoking history (51.1% vs. 43.9%, χ^2^ = 0.791, *p* = 0.374). In contrast, diabetes mellitus was more common in the CAC-negative group (68.3% vs. 47.9%, χ^2^ = 6.471, *p* = 0.011). Given the relatively small sample size of the CAC-negative subgroup, this finding is hypothesis-generating and requires further validation in larger cohorts.

### 3.6. Age-Specific Distribution of CAC and Its Clinical Underutilization

**Figure 7:**
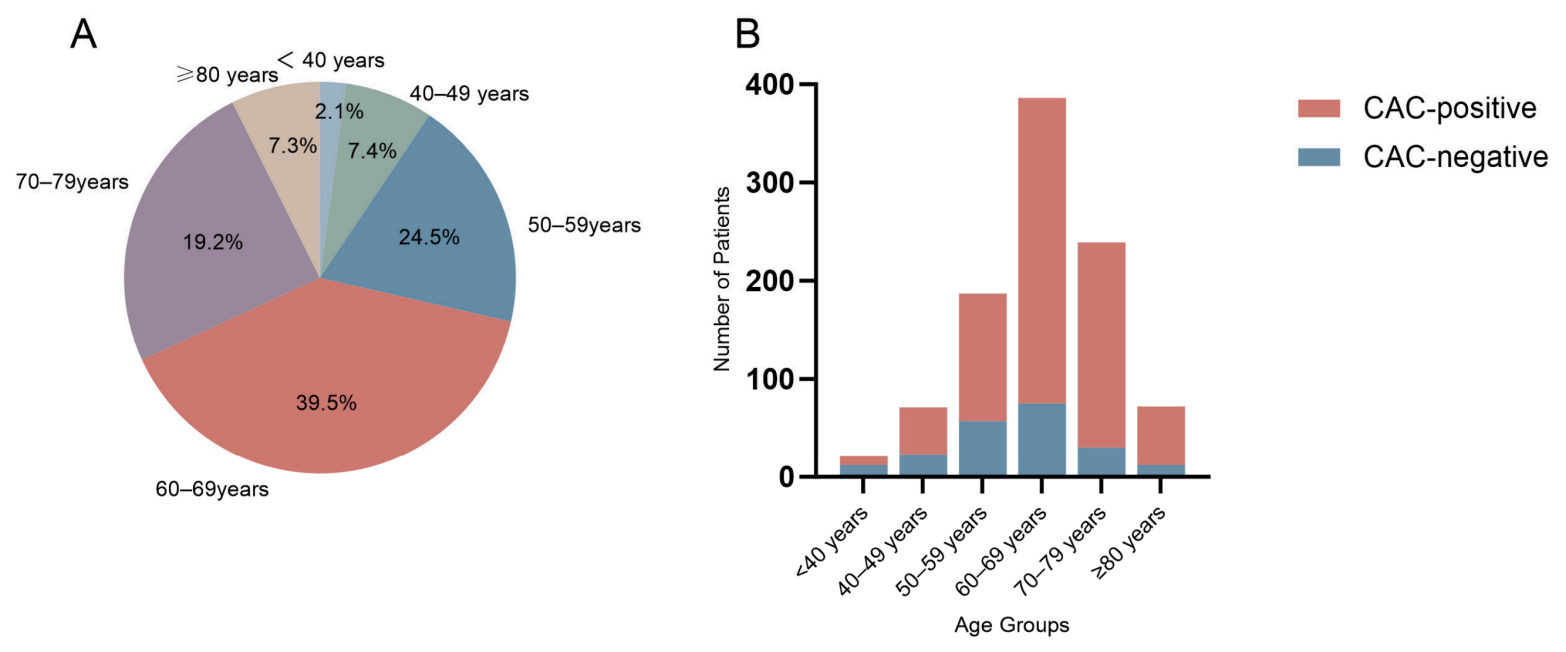
Age distribution of patients undergoing coronary angiography and age-dependent prevalence trend of coronary artery calcification (CAC). (**A**) Pie chart illustrating the age composition of the angiographic cohort. The 60–69-year age group accounted for the largest proportion (39.5%), followed by the 70–79-year group (19.2%). (**B**) Stacked bar graph depicting the case numbers of CAC-positive and CAC-negative subjects across successive age strata. Linear-by-linear association chi-square test confirmed a statistically significant positive correlation between advancing age and CAC positivity (χ^2^ = 37.435, *p* < 0.001).

The population seeking cardiology care, particularly those undergoing coronary angiography (CAG), is predominantly middle-aged and elderly (39.5% aged 60–69 years and 19.2% aged 70–79 years) (Figure 7A). Middle-aged and elderly individuals exhibit a high prevalence of CAC: in our CAG cohort, the positivity rate of CAC increases significantly with age (Figure 7B). Furthermore, analysis of chest CT scans from community-based outpatients without known cardiovascular disease at our institution demonstrated a CAC-positive rate exceeding 50% in patients aged ≥60 years and over 75% in those aged ≥70 years. The near-universal CAC in the elderly impairs its prognostic value for individual risk stratification, and this issue is exacerbated by most cardiology clinic patients being elderly rather than asymptomatic young people. This high prevalence creates a practical barrier: clinicians cannot intervene for all incidental CAC findings. Thus, despite guideline endorsement of CAC utility, its priority in clinical decision-making is inevitably reduced.

## 4. Discussion

The angiographic cohort of the current study predominantly comprised middle-aged and elderly patients, with the 60–69-year age bracket accounting for the largest proportion. The prevalence of coronary artery calcification (CAC) increased linearly with advancing chronological age, a trend concordant with multiple population-based cohort investigations [4,5]. Given the high background prevalence of age-driven degenerative vascular remodeling in older adults, binary CAC positivity alone delivers limited discriminatory capacity for individualized cardiovascular risk stratification among the geriatric population. In contrast, the baseline prevalence of CAC remains exceedingly low in younger and middle-aged individuals; even trivial calcific deposits robustly signal early active atherosclerotic plaque activity and carry strong prognostic alerting value, which aligns closely with the recommendations laid out in the 2026 ACC/AHA blood lipid management guidelines [2].

In routine clinical practice worldwide, standardized CAC quantification using ECG-gated cardiac CT remains underimplemented, particularly among younger age groups. A substantial subset of young and middle-aged patients experience their first atherosclerotic cardiovascular event as acute myocardial infarction without any preceding prodromal manifestations. By contrast, non-targeted routine chest CT is ubiquitously ordered in daily clinical workflows, creating a valuable window for opportunistic coronary artery disease screening through incidental CAC identification. The 2026 ACC/AHA guidelines have formally designated incidental CAC discovered on non-ECG-gated chest CT as objective evidence of subclinical atherosclerosis and mandated its incorporation into cardiovascular risk management decision-making [2]. Real-world clinical practice still suffers from substantial under-recognition and inadequate downstream clinical action for incidental CAC identified on non-ECG-gated chest CT, even when calcification lesions are clearly visible on imaging [6]. In our cohort, a considerable proportion of patients underwent chest CT prior to invasive coronary angiography. Specifically, 55.7% of patients presenting with acute myocardial infarction received chest CT within 12 months preceding angiography, and coronary calcification was radiologically detectable in 93.5% of these subjects. The age-dependent risk signal of CAC positivity raises a potential hypothesis that earlier recognition of sub-clinical coronary calcification in younger individuals may provide valuable preventive opportunities. Together, our observations underscore that unselected chest CT serves as a potent tool for opportunistic CAD risk stratification, offering a potential window for earlier primary-prevention intervention in real-world practice.

The 2026 ACC/AHA guidelines further recognized that validated artificial intelligence algorithms can substitute subjective visual estimation for CAC quantification on non-ECG-gated chest CT, enabling high-throughput, standardized opportunistic screening and mitigating inter-institutional heterogeneity in CAC reporting criteria [2]. Multiple external real-world validation cohorts have demonstrated satisfactory diagnostic performance of non-gated routine chest CT for qualitative dichotomization of CAC presence versus absence, and ordinal categorical CAC burden yields a correlation coefficient of 0.86 when referenced against the gold-standard Agatston score for risk tier allocation [6,7]. Deep learning-facilitated AI pipelines thus offer a viable technical solution for large-scale quantitative opportunistic CAC assessment [8,9,10,11,12], and existing automated CAC scoring algorithms have achieved favorable concordance with risk stratification derived from ECG-gated cardiac CT protocols [13]. Artificial intelligence automatically generates continuous quantitative calcification burden metrics and ordinal severity grades, fundamentally resolving the intrinsic drawbacks of simplistic binary visual categorization. Embedding automated scoring modules into routine radiological workflows can harmonize nationwide CAC reporting standards and potentially remodel the entire framework of opportunistic CAC screening [14]. Several academic societies have released consensus statements regarding CAC reporting on non-ECG-gated chest CT scans, endorsing either quantitative Agatston scoring or qualitative three-tier visual grading (mild/moderate/severe), both of which effectively predict adverse cardiovascular endpoints [14,15]. Several FDA-cleared fully automated AI CAC quantification tools have been deployed in large tertiary medical centers across the United States in recent years. Compared with conventional manual visual assessment, AI-augmented reporting of incidental calcification significantly elevates the standardized prescribing rate of statins and other cardioprotective preventive agents [5]. The rapid uptake of AI-powered opportunistic detection of coronary artery calcification (ODCAC) quantification has compelled healthcare institutions to establish standardized protocols governing CAC documentation, interpretative pathways and subsequent therapeutic linkage.

Multivariate logistic regression analyses demonstrated that incidental CAC detected on unselected chest CT constituted the strongest independent predictor of obstructive coronary artery disease, with an odds ratio of 11.732, outperforming established traditional cardiovascular risk factors including cigarette smoking and diabetes mellitus. Lesion-stratified subgroup analyses revealed that patients with CAC-positive CAD exhibited a higher prevalence of dyslipidaemia, consistent with the canonical pathogenic cascade whereby lipid deposition initiates atherosclerotic plaque formation and subsequent vascular calcification [16]. Conversely, diabetes mellitus was disproportionately prevalent among CAC-negative CAD patients. The underlying pathophysiology resides in diabetic microvascular injury, which predominantly generates non-calcified vulnerable plaques capable of progressing to obstructive coronary stenosis without detectable coronary calcification [3,16]. Clinicians should therefore exercise extreme prudence when interpreting CAC-negative results from thin-slice non-ECG-gated chest CT. Even with ECG-gated coronary CTA—the reference standard for CAC quantification—cases with zero Agatston score but severe luminal obstructive stenosis remain well-documented. A large Danish nationwide registry cohort reported that 58% of symptomatic patients younger than 40 years with obstructive coronary lesions had no measurable CAC, a phenomenon attributed to the predominance of non-calcified vulnerable plaques in early-onset premature atherosclerosis [17]. Our regression models identified active smoking, dyslipidaemia and elevated glycated haemoglobin as secondary independent predictors for obstructive CAD, which exert marked synergistic detrimental effects.

From a pragmatic perspective, large-scale clinical rollout of AI-assisted quantitative CAC measurement continues to face multiple practical implementation barriers. Prior to the universal adoption of automated AI CAC scoring systems within radiology departments, it remains imperative to formulate rational strategies to guide clinical decision-making relying solely on binary CAC results yielded by routine chest CT. Age acts as an unavoidable confounding covariate when applying crude CAC presence/absence dichotomization. Given the very low baseline CAC prevalence in younger populations, any identifiable calcification confers strong predictive power for underlying obstructive CAD. Of note, all CAC-positive patients aged below 40 years within our angiographic cohort were ultimately diagnosed with obstructive CAD; however, the limited sample size within this young age stratum restricts the external validity of this observation, mandating further validation in larger-scale prospective cohorts. On the basis of the core tenet from the 2026 ACC/AHA guidelines that age-specific 75th percentile CAC burden constitutes a Class I indication for statin initiation, together with the epidemiological pattern of progressive CAC positivity with advancing age in the general population, we propose an exploratory practical algorithm. First, an age cutoff corresponding to 25% community-level CAC positivity can be defined. Population-based sex-stratified age thresholds for urban Chinese cohorts have recently been published, with the 25% CAC-positivity boundary at 46 years for men and 56 years for women [18]. For subjects younger than this threshold, where population-wide CAC positivity stays below 25%, any detectable CAC can be regarded as equivalent to age-matched 75th percentile calcific burden, thus meeting the indication for statin initiation.

Second, a separate age threshold can be designated for elderly cohorts. Above this cutoff, isolated CAC negativity becomes relatively infrequent in the general population, allowing risk de-escalation for multimorbid older adults on polypharmacy regimens to avoid statin overprescription and overtreatment. Existing evidence only confirms that adults aged ≥75 years with an Agatston score of zero achieve excellent long-term survival, with a 98% survival rate at 5.6 years and classification into the extremely low cardiovascular risk stratum [19]. Nevertheless, the population proportion of CAC-negative individuals aged ≥75 years and their quantitative correlation with incident coronary heart disease events remain insufficiently characterized, which could serve as a direction for subsequent targeted research. It should be emphasised that this exploratory age-percentile-based interpretation algorithm has not been prospectively validated in our cohort, and the present findings only provide indirect evidence to support this conceptual framework. Importantly, formal diagnostic-performance metrics including AUC, sensitivity, specificity, PPV, and NPV were not calculated for individual age-stratified subgroups in the present analysis. Our age-dependent interpretative framework is derived from a combination of the conditional prevalence observed in our cohort and existing clinical-practice guideline recommendations.

While advancing age is an independent risk factor for coronary-artery calcification and calcification burden rises progressively with age, CAC yields reduced incremental value for risk stratification of obstructive coronary artery disease among older adults. This decline in discriminative performance does not mean that CAC loses its clinical utility. In younger individuals, visual detection of incidental coronary calcification on non-ECG-gated chest CT can serve as a convenient preliminary risk indicator. By contrast, simple visual assessment is insufficient in older adults with high baseline calcification burden. Formal Agatston scoring with reference to age- and sex-specific 75th percentile thresholds is therefore required to achieve reliable risk stratification in this elderly population. Elevated calcification load alone cannot reliably indicate the presence of obstructive stenosis among older patients, which explains the reduced incremental benefit of CAC-based risk stratification.

The present investigation was conducted as a single-center retrospective observational study, which inherently carries unavoidable selection bias. Notably, our study cohort only included patients referred for invasive coronary angiography, which may introduce referral bias. Accordingly, our findings should not be directly extrapolated to general screening populations. A substantial proportion of otherwise-eligible participants (42%, *n* = 771) were excluded due to the absence of pre-procedural non-ECG-gated chest CT. Potential systematic differences in clinical acuity, pulmonary comorbidities and hospitalisation route may exist between included and excluded individuals, which may further introduce selection bias. Since the enrolled study population mainly consisted of hospitalized patients who were referred for invasive coronary angiography for suspected coronary artery disease, the overall prevalence of coronary artery calcification derived from this cohort is likely to overestimate the true CAC burden in the general unselected population. Although the total sample size of 976 participants was statistically adequate to complete analyses focused on primary research endpoints, several subgroup analyses stratified by age, lesion characteristics and comorbidities suffered from relatively small sample volumes, leading to insufficient statistical power to detect modest or subtle intergroup differences that may carry clinical implications. Furthermore, baseline long-term medication data on statins and aspirin before admission were missing for some patients. Information on other important covariates, including dietary patterns, family history of atherosclerotic cardiovascular disease, and the duration of diabetes mellitus, was also incomplete. These unrecorded factors can influence both systemic lipid metabolism, vascular calcification progression and the risk of obstructive coronary stenosis, and this missing information may introduce residual confounding bias into the analytical associations between concomitant metabolic diseases and CAC status, potentially leading to over-estimation of the observed odds-ratio. In addition, CAC was assessed using an ordinal visual grading system rather than the reference-standard Agatston score. The ordinal grading approach may be less sensitive and less precise than Agatston-based quantitative scoring, which could affect the detection and risk classification of coronary calcification. Finally, the number of young individuals under 40 years of age was relatively limited within our dataset. While theoretical reasoning suggests that the cardiovascular preventive benefit triggered by incidental CAC detection on routine chest CT is most prominent in younger patients, larger-scale prospective multicenter cohorts are still required to externally validate the reliability and clinical utility of CAC-based risk stratification among early-onset atherosclerotic populations.

## 5. Conclusions

Incidentally detected coronary artery calcification on non-ECG-gated routine chest CT serves as a robust independent predictor for obstructive coronary artery disease, yet its predictive performance varies substantially across different age strata largely due to progressive age-related degenerative vascular remodeling. Artificial intelligence-enabled automated CAC quantification derived from opportunistic non-gated chest CT demonstrates favorable correlation with the reference-standard Agatston score, which offers a feasible approach to standardize CAC reporting across radiological departments and overcome the inherent limitations of simple binary visual interpretation alone. Concurrent smoking exposure combined with positive CAC yields synergistically elevated risk for obstructive coronary lesions, reinforcing the necessity of intensive multifactor risk reduction interventions for this ultra-high-risk subgroup of patients. More importantly, the age-stratified interpretative algorithm incorporating age-specific CAC percentile thresholds recommended by the latest guidelines is clinically operable in daily practice, which not only provides standardized evidence for statin initiation but also helps avoid unnecessary overtreatment among low-risk elderly patients with negative CAC results on thoracic computed tomography. Collectively, age-calibrated opportunistic CAC screening supported by artificial intelligence may represent a pragmatic and evidence-based strategy to improve cardiovascular primary-prevention management among patients undergoing chest CT within routine clinical care. Its performance among unselected asymptomatic community-dwelling populations remains to be confirmed in further dedicated studies. Further multi-center validation and implementation studies focusing on younger individuals are still required before broader clinical application.

## Figures and Tables

**Table 1:** Baseline characteristics of 976 patients.

Variables	CAC-Positive Group (*n* = 773)	CAC-Negative Group (*n* = 203)	Statistical Value (*p*)
Demographics			
Age, years	65.63 ± 10.36	60.23 ± 12.07	<0.001
Male, *n* (%)	537 (69.5%)	106 (52.2%)	<0.001
BMI, kg/m^2^	25.81 ± 3.79	26.01 ± 3.83	0.51
Medical History, *n* (%)			
Hypertension	523 (67.7%)	118 (58.1%)	0.011
Diabetes Mellitus	355 (45.9%)	49 (24.1%)	<0.001
Dyslipidemia	565 (73.1%)	108 (53.2%)	<0.001
Smoking History	359 (46.4%)	57 (28.1%)	<0.001
Laboratory Parameters			
FPG, mmol/L	6.30 ± 2.24	5.80 ± 1.79	0.001
HbA1c, %	6.52 ± 1.36	6.14 ± 1.45	0.001
LDL-C, mmol/L	2.62 ± 0.93	2.58 ± 0.77	0.529
TG, mmol/L	1.68 ± 1.06	1.61 ± 1.11	0.393

Normally distributed continuous variables were compared by independent-samples *t*-test. Categorical variables were analysed using the Pearson χ^2^ (chi-square) test. All statistical tests were two-sided.

**Table 2:** Prevalence and odds ratios for coronary stenosis ≥ 50% and ≥70% across CAC grade subgroups.

CAC Grade	Cases (*n*)	Coronary Stenosis ≥ 50% [*n*(%)]	OR (95%CI)	Coronary Stenosis ≥ 70% [*n*(%)]	OR (95%CI)	*p*-Value
Grade 0 *	203	41 (20.2)	1	34 (16.7)	1	—
Grade 1	248	156 (62.9)	6.700 (4.364–10.286)	117 (47.2)	4.439 (2.845–6.926)	<0.001
Grade 2	208	169 (81.3)	17.122 (10.505–27.907)	133 (63.9)	8.815 (5.539–14.027)	<0.001
Grade 3	317	278 (87.7)	28.165 (17.440–45.485)	233 (73.5)	13.787 (8.836–21.513)	<0.001

*: CAC Grade 0 served as the reference group for odds ratio calculation.

**Table 3:** Adjusted Odds Ratios of Independent Predictors for coronary artery disease.

Variable	Adjusted Odds Ratio	Lower 95% CI	High 95% CI	*p*-Value
Coronary Artery Calcification	11.732	7.700	17.875	<0.001
Smoking History	3.102	2.093	4.597	<0.001
Dyslipidemia	1.519	1.045	2.209	0.028
Glycated Hemoglobin (HbA1c)	1.300	1.052	1.607	0.015
Age (years)	1.013	0.996	1.03	0.142
Sex (Male)	1.084	0.733	1.602	0.686
LDL-C (mmol/L)	1.151	0.944	1.402	0.165
Triglycerides (TG, mmol/L)	0.926	0.770	1.112	0.410
Fasting Plasma Glucose (mmol/L)	1.037	0.910	1.181	0.587
Hypertension	0.753	0.522	1.087	0.13
Diabetes Mellitus	1.431	0.908	2.254	0.122
BMI (kg/m^2^)	1.001	0.954	1.049	0.976

## Data Availability

The datasets generated and analyzed during this retrospective study cannot be publicly released due to patient-privacy-related ethical restrictions from the Institutional Review Board. The de-identified minimal dataset supporting the findings is available from the corresponding author upon reasonable request.

## Supplementary Materials

The following supporting information can be downloaded at https://www.mdpi.com/article/10.3390/jcdd13090471/s1. Table S1: Distribution of CAC grades across five predefined age strata among the overall cohort (n = 976).
