# Supplementary material for: Age-Dependent Efficacy of Incidental Coronary Artery Calcium on Pre-CAG Chest CT: Potentially Missed Prevention Opportunities in Chinese Population
Source: J Cardiovasc Dev Dis. 2026 Sep 20;13(9):471. doi: 10.3390/jcdd13090471 (PMC13607687; doi:10.3390/jcdd13090471)
Supplement: Supplementary file 1 [file jcdd-13-00471-s001.zip › jcdd-4541941-supplementary.pdf]

Table S1. Distribution of CAC grades across five predefined age strata among the overall cohort (n=976)

Age group	Total n	CAC-positive n (%)	Grade 0 n (%)	Grade 1 n (%)	Grade 2 n (%)	Grade 3 n (%)
29-39 years	21	9 (42.9%)	12 (57.1%)	6 (28.6%)	3 (14.3%)	0 (0.0%)
40-49 years	71	48 (67.6%)	23 (32.4%)	36 (50.7%)	9 (12.7%)	3 (4.2%)
50-59 years	187	133 (71.1%)	54 (28.9%)	45 (24.1%)	37 (19.8%)	51 (27.3%)
60-69 years	386	314 (81.3%)	72 (18.7%)	101 (26.2%)	84 (21.8%)	129 (33.4%)
≥70 years	311	269 (86.5%)	42 (13.5%)	60 (19.3%)	75 (24.1%)	134 (43.1%)

Footnote: Grade 0 = no coronary calcification (CAC-negative); Grade 1-3 represent mild, moderate and severe CAC, respectively. CAC grade was derived from summed Shemesh ordinal scores (range 0-12) across four major coronary arteries. Percentages are row-percentages within each age stratum.
